# Opioid use disorder: current trends and potential treatments

**DOI:** 10.3389/fpubh.2023.1274719

**Published:** 2024-01-25

**Authors:** Yu Kyung Lee, Mark S. Gold, Kenneth Blum, Panayotis K. Thanos, Colin Hanna, Brian S. Fuehrlein

**Affiliations:** ^1^Department of Psychiatry, Brigham and Women’s Hospital, Boston, MA, United States; ^2^Department of Psychiatry, Washington University in St. Louis Euclid Ave, St. Louis, MO, United States; ^3^Division of Addiction Research and Education, Center for Sports, Exercise, and Mental Health, Western University Health Sciences, Pomona, CA, United States; ^4^Behavioral Neuropharmacology and Neuroimaging Laboratory on Addictions, Department of Pharmacology and Toxicology, Jacobs School of Medicine and Biosciences, Clinical Research Institute on Addictions, State University of New York at Buffalo, Buffalo, NY, United States; ^5^Department of Psychiatry, Yale University, New Haven, CT, United States

**Keywords:** opioid-related disorders, public health, therapeutics, electrical stimulation of the brain, hallucinogens, neuroimmunomodulation, pro-dopamine-regulation, homeostasis

## Abstract

Opioid use disorder (OUD) is a major public health threat, contributing to morbidity and mortality from addiction, overdose, and related medical conditions. Despite our increasing knowledge about the pathophysiology and existing medical treatments of OUD, it has remained a relapsing and remitting disorder for decades, with rising deaths from overdoses, rather than declining. The COVID-19 pandemic has accelerated the increase in overall substance use and interrupted access to treatment. If increased naloxone access, more buprenorphine prescribers, greater access to treatment, enhanced reimbursement, less stigma and various harm reduction strategies were effective for OUD, overdose deaths would not be at an all-time high. Different prevention and treatment approaches are needed to reverse the concerning trend in OUD. This article will review the recent trends and limitations on existing medications for OUD and briefly review novel approaches to treatment that have the potential to be more durable and effective than existing medications. The focus will be on promising interventional treatments, psychedelics, neuroimmune, neutraceutical, and electromagnetic therapies. At different phases of investigation and FDA approval, these novel approaches have the potential to not just reduce overdoses and deaths, but attenuate OUD, as well as address existing comorbid disorders.

## Introduction

Opioid use disorder (OUD) is a complex physical and emotional disease with important mood and anhedonic impacts. It also contributes to comorbid medical and infectious diseases that require evaluation and treatment (1). Drug use changes the brain, behavior, and motivational hierarchy via induction of neuroplasticity; the patient with OUD acquires a chronic, progressive neurodysregulation that shortens life, reduces career opportunities and earning potential, increases the risks of other diseases, and often ends in death. Fentanyl is becoming increasingly common. Fentanyl is highly effective at producing OUD and contributing to anhedonia and overdose. Many with OUD also use methamphetamine and cocaine for their euphoric mood effects, but when used regularly, these drugs can lead to alterations in brain function to trigger negative effects, such as dysphoria, anhedonia, and depression (2). Unfortunately, polysubstance use and relapses are the norm rather than the exception (3, 4). OUD is part of the substance use disorder (SUD) spectrum but is unique among other SUDs in that there is a solid understanding of its neurobiology and there are Food and Drug Administration (FDA) approved treatments (5). Medical management of OUD has not changed much since the 1970s, with agonists like methadone and antagonists like naltrexone. The addition of the partial agonist buprenorphine and extended-release naltrexone (XR-NTX) were important milestones.

## Opioid use in the context of worsening substance use

Substance use disorder (SUD) is one of the nation’s most pressing public health challenges. According to the 2021 World Drug Report by the United Nations Office on Drugs and Crime (UNODC), at least 275 million people worldwide used controlled substances in the previous year, with more than 36 million meeting the criteria for a substance use disorder (6). Among controlled substances contributing to the burden of disease, opioids stand out as the primary driver of drug-related fatalities, comprising 69% of deaths directly associated with drug use.

Recent data show the evidence-based and FDA-approved treatments for OUD reduce stigma and improve treatment access. Although more people are currently being treated with medications for opioid use disorder (MOUD), deaths continue to increase. Nationally, over the past 15 years, at least 500,000 deaths have been attributed to opioid overdoses, contributing to the decrease in US life expectancy. This concerning trend has worsened during the COVID-19 pandemic (7, 8). Those with SUDs are highly dependent on traditional in-person and often emergent care (9). However, pandemic policies--such as the quarantine--to save lives from the COVID-19 virus, have led to disruptions in such care, leading to worsening opioid overdoses and deaths during the lockdown (10, 11). The Centers for Disease Control and Prevention estimated that for the first time ever, over 100,000 deaths occurred due to overdoses during a 12-month period, and the current Director of the Office of National Drug Control Policy suggested that annual opioid-induced deaths could reach 165,000 by 2025 (12).

By necessity, the opioid crisis has shifted the focus to addressing overdose deaths rather than treatment and recovery. A considerable proportion of opioid overdose fatalities are now linked to synthetic opioids, particularly fentanyl. The opioid epidemic has transitioned from being primarily of prescription opioids to heroin and now subsequently fentanyl. Concerningly, with the increase in synthetic opioid use, the trend shows that more and more individuals are also consuming other substances, sometimes inadvertently from contamination and others from co-ingestion of other prescription or psychostimulant drugs. Although SUDs are often discussed in isolation, the reality is that many individuals are combining multiple drugs, often in fatal combinations. For example, in more than half of all methamphetamine-related deaths and about three-quarters of all cocaine-related deaths in 2019, there were co-ingestion of opioids (7). Illicitly manufactured fentanyl is implicated in the increase in overdose deaths in cocaine use, and co-ingestion of fentanyl and other substances, such as methamphetamine, cocaine, and ecstasy, have been shown in postmortem examinations of overdose deaths (13, 14). Patients in the psychiatric emergency rooms often test positive for fentanyl while testing positive for other psychostimulants; unknown contamination with fentanyl puts opioid-naïve psychostimulant users at an increased risk of overdose (15, 16). While the public attention remains on opioid-related deaths, a concerning upsurge in fatalities linked to stimulant drugs suggests that the opioid crisis may be entering a new phase. The ongoing substance use crisis is constantly evolving, marked by changing patterns of substance use and availability, as well as concurrent use of multiple drugs across drug classes.

## OUD overdose reversal starts with naloxone

Among all potential interventions, increasing the access to naloxone would have the most significant effect in reducing opioid-use related deaths, according to Pitt et al. (17). While important to implement other strategies, no other harm reduction approach has had as significant of an impact. Naloxone is classified as a “pure” antagonist, meaning it lacks opioid agonistic traits seen in other opioid antagonists, and it displaces other full and/or partial opioid agonists that engage opioid receptors to reverse the effects of euphoria, analgesia, as well as respiratory depression, sedation, and bradycardia. Naloxone is a rapid-acting, easy-to-administer agent that can be given in the setting of opioid intoxication and overdose, to provide swift, life-saving reversal (18). Within minutes, naloxone can fully reverse the effects of opioids. Regardless of the substance use history, naloxone offers substantial potential benefits and minimal risk when overdose is suspected. Thus, the importance of promoting access to naloxone in those who use opioids as well as in potential bystanders who can intervene in an overdose setting cannot be understated. However, it is also important to note that naloxone serves as an intervention rather than a remedy for the underlying condition. Naloxone can treat the overdose acutely, but it does not treat the OUD. Existing evidence does not indicate that experiencing an overdose and subsequent reversal with naloxone alters the trajectory of those with OUD; patients must be connected to subsequent treatment services that include MOUDs for improved outcomes (1). Thus, access to both is critical to reduce OUD related deaths.

## Transition with urgency: from overdose reversal to treatment of the whole patient with OUD

It is difficult to accurately estimate the total economic burden associated with substance use, encompassing factors from the cost of treatment as well as reduced productivity, loss of life, and the emotional toll on those left behind (19). Although it has been suggested that mortality can be reduced by evidenced-based treatment approaches, OUD is characterized by a chronic and relapsing course—initially driven by activation of the brain’s reward system, but later increasingly dominated by anti-reward neural circuits that trigger negative emotional states and relapses (3, 20). It is noteworthy that one important anti-reward neurocircuit phenomena is the subsequent opioid-induced reduction of functional connectivity (21).

No single treatment approach has proven to be universally effective, as many have other medical and psychiatric comorbidities that often hinder successful treatment. Furthermore, racial and economic disparities in morbidity and mortality point to inequalities that must be addressed and rectified (22). Even harm reduction strategy of naloxone is a limited strategy in that it often requires someone else other than the patient to help administer the reversal agent (23).

The current mainstay of treatment for OUD is MOUDs. Buprenorphine, extended-release naltrexone (XR-NTX), and methadone are all FDA-approved and have been shown to be effective in reducing the number of overdoses in those who remain adherent to treatment—which remains a challenge in access and retention (24–26). Buprenorphine is the most prescribed of the MOUDs and it may help mitigate anhedonia and withdrawal symptoms associated with fentanyl use (27, 28). However, treatment dropouts are common, and patients are often re-initiated on the same buprenorphine (29). Once an overdose is reversed, the patient remains at high risk for overdose, which has led to initiation of buprenorphine in hospitals, emergency rooms, and immediately after rescue. This has not decreased the overall number of deaths, but provides another opportunity to successfully treat the patient.

XR-NTX is a monthly injectable opioid antagonist that acts to block other opioids from activating receptors. Despite the monthly injectable formulation of the drug, transition and compliance to treatment remain an issue, as patients need to go through detoxification to start the medication. Therefore, despite similar efficacy of XR-NTX to buprenorphine shown in the XBOT trial, buprenorphine is more often initiated (30). Augmentation of naltrexone therapy with other agents such as clonidine, lofexidine, and buprenorphine are often tried in outpatient detoxification settings. With augmentation of non-opioid agents like clonidine, patients are able to successful initiate and maintain an opioid antagonist, rather than a partial agonist. This is particularly important for those in occupations related to public for whom methadone or buprenorphine is not prescribed and for those who may be mandated to treatment for their OUD (26, 31).

While MOUDs are highly effective and FDA approved, they are not a “magic bullet”, and the entire scientific community should continue their pursuit to develop alternative non-addictive and safe treatments (32, 33). Despite best efforts, the existing strategies for preventing and treating OUD will still result in more than 700,000 deaths in the US between 2016 and 2025, from both prescription and non-prescription opioids such as fentanyl (34).

The most favorable outcomes are associated with extended duration of MOUD therapy. However, treatment drop out and relapses are common. When OUD treatment with a MOUD ends or is discontinued by the patient, they often relapse, overdose, and rarely have the disease remitted. Risk of overdose appears to persist even after completion of buprenorphine treatment, and stopping MOUD is associated with an elevated overdose risk, raising a controversial question about whether opioid agonist treatment can lead to opioid deficiency or opioid system dysregulation (3, 35). This risk of relapse and overdose persists even several years of recovery; thus, recovery can be akin to a “remission” of OUD symptoms rather than a complete cure or elimination. While it may be true that prolonged MOUD is the most effective in preventing relapse, it is also true that the quality of life on MOUD treatment can be negatively impacted and most patients do not remain on lifelong treatment for OUD. One study revealed that patients on long-term Suboxone exhibited significantly flat affect (*p* < 0.01) and reported diminished sense of feelings of happiness, sadness, and anxiety compared to both the general population and Alcoholics Anonymous (AA) groups (36). Despite the limitations, treatment of OUD is still linked to improved outcomes in morbidity and mortality, and integration of MOUD with effective, evidence-based therapy and contingency management lead to enhanced outcomes (37). It is thought that achieving successful treatment of OUD requires not just medications but a comprehensive approach that addresses the psychosocial factors that predispose and perpetuate individuals toward opioid use disorder.

Brain and behavioral recovery take hope, patience, time, and effort. No one knows if or when the brain will return to its pre-morbid function. Interestingly, research from China revealed that even after 10 months of heroin abstinence, there were changes in resting state of functional connectivity (RSFC) patterns between the midbrain and various cortical regions, such as diminished RSFC of the medial orbitofrontal cortex (mOFC) and anterior cingulate cortex compared to non-heroin using controls. Persistent reward circuitry abnormalities were present after 16 months, but enhancement of RSFC in certain circuits were seen in long-term abstinence compared with short-term (38, 39). Abstinence from substance use, as well as adopting a healthy diet, and other regenerative treatments, including exercise and transcranial magnetic stimulation may all help expedite brain recovery (40, 41).

## Pro-dopamine regulation and assessment of preaddiction

At the population health level, a “preaddiction” model, like prediabetes, has been suggested to ring the alarm bell for an early intervention before the addiction progresses to cause more severe symptoms and engender chronic changes in the brain’s neural circuitry. SUD is currently defined by the DSM-5 based on 11 symptoms of impaired control, and severity is determined by the number of symptoms patients endorse. The term addiction specifically refers to severe SUD, which is defined as having six or more symptoms. This occurs in about 4–5% of adults, compared to 13% of the adult population who have mild to moderate SUD, defined as having 2–5 symptoms (42). Although larger proportion of the population suffer from mild to moderate SUD, public health policies and treatment focus on those with severe, often chronic addictions, to prevent overdoses and deaths, rather than the much larger population grappling with early-stage SUDs. By focusing on those with early-stage addiction, McLellan et al. argue that a preaddiction model that looks for early signs of addiction increases public awareness and allows early intervention that can increase disease detection, shorten delays to treatment, and prevent progression (42, 43). Directors of the National Institute on Drug Abuse and National Institute on Alcohol Abuse and Alcoholism have also advocated for the integration of “preaddiction” to the conceptualization of addiction in the DSM.

Although the term preaddiction borrows from the advances made in diabetes, but it is best conceptualized as dopamine or reward dysregulation, where there is a net hypodopaminergia within the meso-limbic reward circuitry from inappropriate or dysregulated neurotransmitter systems (44, 45). Therefore, the terms “reward deficiency” or “reward dysregulation” have also been proposed (46, 47). Reward deficiency syndrome (RDS) refers to behavioral dysfunctions resulting from the disruption of the reward circuitry, due to both genetic and epigenetic factors, and can describe a wide spectrum of psychiatric disorders, from various addictions to obsessive and compulsive disorders and other behavioral conditions (47).

## Ways to improve on existing treatment approaches

Public health solutions proposed to address the worsening opioid epidemic include: relaxing the restrictions for physicians to be able to prescribe MOUD; increasing insurance coverage to increase access to treatment for patients (48–51); inducting patients on high-dose buprenorphine in the emergency department (52, 53); and implementing contingency management along with medical treatment to address other key issues of patients undergoing treatment, such as other concurrent substance use (50). Widespread adoption of these practices may help improve treatment retention and reduce overdoses and deaths, to address critical gaps in delivery of existing treatments (37, 54–56).

All these measures would be helpful to curb damage of the OUD at the population level, but still does not address the intrinsic limitations of existing treatments. Limited effectiveness of existing pharmacologic treatments and harm reduction approaches leaves a desire for more durable, novel treatment approaches especially as they relate to the required induction of “dopamine homeostasis” (57–59).

Novel treatments for SUDs are being investigated, most notably interventional neuromodulatory interventions such as transcranial magnetic stimulation (TMS) and deep brain stimulation (DBS), as well as psychedelics and neuroimmune therapies. Rather than working at modulating specific opioid receptors as existing MOUDs do, these proposed treatments target other aspects of substance use pathophysiology, such as the mesolimbic neural circuitry modulating reward and dorsolateral prefrontal and orbitofrontal cortex involved in craving, or the neuroimmune and epigenetic modulations that occur in response to substances (60, 61).

## Developments in interventional neuromodulation treatments for OUD

Interventional neuromodulational treatments such as TMS and DBS work by modulating neural circuits and synaptic plasticity, and several neuromodulating modalities have been investigated for use in various neurologic and psychiatric conditions (62). TMS is a noninvasive treatment that utilizes an extracranial magnetic coil to induce a magnetic field that can stimulate or inhibit targeted cortical and subcortical structures. It is currently FDA-approved for treatment of psychiatric conditions including major depressive disorder and obsessive-compulsive disorder. Clinical trials have shown its effectiveness in reducing substance craving via targeting of the dorsolateral prefrontal cortex, with effects on curbing substance use lasting greater than the immediate treatment period, with various substances such as cocaine, methamphetamine, alcohol, heroin, and cigarette use (63–70). A meta-analysis of repetitive TMS (rTMS) on addiction has found a small, persistent effect on stimulant and behavioral addiction, but not on depressants such as alcohol, opioids, and cannabis (71). However, currently multiple randomized controlled trials are being conducted for various TMS protocols to treat patients with opioid use as well as uncover mechanisms (72–83). Further understanding of the exact mechanism and optimization is needed to implement TMS as treatment for OUD (84). One important aspect regarding TMS relates to the concept of utilizing QEEG to determine individualized or personalized signatures termed PRTMS, first coined and developed by Kevin T. Murphy (85).

Similarly, DBS is a neuromodulatory treatment method that can directly stimulate targeted brain regions, but unlike TMS, it typically relies on surgically implanted electrodes. It has FDA approval for various neurologic conditions, such as Parkinson’s disease, tremors, dystonia, as well as epilepsy and OCD. With its ability to reach deeper subcortical structures, DBS is also being investigated for other psychiatric conditions, such as treatment-resistant depression, pain, multiple sclerosis, and addiction. Specifically for addiction, animal models have shown that modulation of nucleus accumbens (NAc) can reverse synaptic changes from cocaine and alcohol use (60, 62), and small case studies in humans have shown effectiveness in treating alcohol use and heroin use (86). More registered clinical trials are examining DBS’s effect in various substance use, including both randomized and non-randomized clinical trials on DBS of NAc of the mesolimbic system as well as DBS specifically for the treatment for severe OUD (87–94).

Beyond TMS and DBS, other similar modalities such as vagus nerve stimulation (VNS), focused ultrasound (FUS), and transcranial direct current stimulation (tDCS) are being investigated, with varying degrees of invasiveness and spatial resolution. VNS is FDA-approved for treatment-resistant depression and epilepsy, and FUS is approved for use in treatment-resistant Parkinson’s disease as well as essential tremor (95, 96), while tDCS does not have any FDA-approved indications. A small randomized clinical trial on transcutaneous VNS has found significant improvement in behavioral and physiologic opioid withdrawal symptoms, indicating potential utility as adjunct treatment for OUD (93). Although not FDA-approved, tDCS has some evidence for utility in depression and addiction, and multiple clinical trials are ongoing for its use in neuropsychiatric conditions (81, 97–101). FUS is also being investigated in a small, open-label clinical trial for feasibility in treatment for OUD (102).

Overall, more evidence is needed before neuromodulatory modalities are adopted in OUD treatment, but the strength of these proposed therapies is that they work via modulating neural circuitries and synaptic plasticity, rather than maintaining patients on opioid maintenance treatments, as well as treat other comorbid neuropsychiatric conditions, such as other SUDs and mood disorders.

## Renewed interest in psychedelics for SUD

Psychedelic medicine has seen a resurgence of interest in recent years as potential therapeutics, including for SUDs (103, 104). Prior to the passage of the Controlled Substance Act of 1970, psychedelics had been studied and utilized as potential therapeutic adjuncts, with anecdotal evidence and small clinical trials showing positive impact on mood and decreased substance use, with effect appearing to last longer than the duration of use. Many psychedelic agents are derivatives of natural substances that had traditional medicinal and spiritual uses, and they are generally considered to have low potential for dependence and low risk of serious adverse effects, even at high doses. Classic psychedelics are agents that have serotonergic activity via 5-hydroxytryptamine 2A receptors, whereas non-classic agents have lesser-known neuropharmacology. But overall, psychedelic agents appear to increase neuroplasticity, demonstrating increased synapses in key brain areas involved in emotion processing and social cognition (105–109). Being classified as schedule I controlled substances had hindered subsequent research on psychedelics, until the need for better treatments of psychiatric conditions such as treatment resistant mood, anxiety, and SUDs led to renewed interest in these agents.

Of the psychedelic agents, only esketamine—the S enantiomer of ketamine, an anesthetic that acts as an NMDA receptor antagonist—currently has FDA approval for use in treatment-resistant depression, with durable effects on depression symptoms, including suicidality (110, 111). Ketamine enhances connections between the brain regions involved in dopamine production and regulation, which may help explain its antidepressant effects (112). Interests in ketamine for other uses are expanding, and ketamine is currently being investigated with plans for a phase 3 clinical trial for use in alcohol use disorder after a phase 2 trial showed on average 86% of days abstinent in the 6 months after treatment, compared to 2% before the trial (113).

Psilocybin, an active ingredient in mushrooms, and MDMA, a synthetic drug also known as ecstasy, are also next in the pipelines for FDA approval, with mounting evidence in phase 2 clinical trials leading to phase 3 trials. Psilocybin completed its largest randomized controlled trial on treatment-resistant depression to date, with phase 2 study evidence showing about 36% of patients with improved depression symptoms by at least 50% at 3 weeks and 24% experiencing sustained effect at 3 months after treatment, compared to control (114). Currently, a phase 3 trial for psilocybin for cancer-associated anxiety, depression, and distress is planned (115). Similar to psilocybin, MDMA has shown promising results for treating neuropsychiatric disorders in phase 2 trials (116), and in 2021, a phase 3 trial showed that MDMA-assisted therapy led to significant reduction in severe PTSD symptoms, even when patients had comorbidities such as SUDs; 88% of patients saw more than 50% reduction in symptoms and 67% no longer qualifying for a PTSD diagnosis (117). The second phase 3 trial is ongoing (118).

With mounting evidence of potential therapeutic use of these agents, FDA approval of MDMA, psilocybin, and ketamine can pave the way for greater exploration and application of psychedelics as therapy for SUDs, including opioid use. Existing evidence on psychedelics on SUDs are anecdotally reported reduction in substance use and small clinical cases or trials (119). Previous open label studies on psilocybin have shown improved abstinence in cigarette and alcohol use (120–122), and a meta-analysis on ketamine’s effect on substance use showed reduced craving and increased abstinence (123). Multiple open-label as well as randomized clinical trials are investigating psilocybin, ketamine, and MDMA-assisted treatment for patients who also have opioid dependence (124–130). Other psychedelic agents, such as LSD, ibogaine, kratom, and mescaline are also of interest as a potential therapeutic for OUD, for their role in reducing craving and substance use (104, 131–140).

## Potential neuroimmune modulatory approaches to treating OUD

Changes to brain’s synaptic plasticity via neuroimmune modulation seen in addiction suggest the role of agents that can disrupt and/or mitigate the changes from substance use and addiction as potential therapeutics (141, 142). Existing medications may be applicable to OUD and substance use treatment in this manner, by modulating or reversing synaptic plasticity. For example, N-Acetylcysteine, an antioxidant medication with multiple clinical applications, has shown to reverse cocaine-induced metaplasticity in in rats (143), and ceftriaxone, a third generation cephalosporine antibiotic, has shown to downregulate glutamate transporter 1 (EEAT2) and reduce drug seeking behavior (144, 145). Similarly, spironolactone, a mineralocorticoid receptor antagonist, has been shown to reduce alcohol consumption in both mice and humans, but to a greater degree in humans (146). Novel immune-modulating agents can also be developed to target known signaling pathways involved in addiction and OUD, such as TLR4, a transmembrane protein that plays a role in rewarding effects of substances, leading to reinforcement of use (147–149).

Other neuroimmune modulatory agents are of interest, including biologic agents, such as antibodies, and vaccines that block the effect of substances of abuse (150–154), but low immunogenicity remains a major challenge to being effective in humans. Overall, much more evidence is needed to develop novel neuroimmunotherapeutics as effective treatments for SUDs. However, the potential benefit of the proposed approaches is that they may modulate or even reverse the lasting damages substance use imposes on the brain.

## Summary

The nation has had a series of drug overdose epidemics, starting with prescription opioids, moving to injectable heroin and then fentanyl. Addiction policy experts have suggested a number of policy changes that increase access and reduce stigma along with many harm reduction strategies that have been enthusiastically adopted. Despite this, the actual effects on OUD & drug overdose rates have been difficult to demonstrate.

The efficacy of OUD treatments is limited by poor adherence and it is unclear if recovery to premorbid levels is even possible. Comorbid psychiatric, addictive, or medical disorders often contribute to recidivism. While expanding access to treatment and adopting harm reduction approaches are important in saving lives, to reverse the concerning trends in OUD, there must also be novel treatments that are more durable, non-addicting, safe, and effective. Promising potential treatments include neuromodulating modalities such as TMS and DBS, which target different areas of the neural circuitry involved in addiction. Some of these modalities are already FDA-approved for other neuropsychiatric conditions and have evidence of effectiveness in reducing substance use, with several clinical trials in progress. In addition to neuromodulation, psychedelics has been gaining much interest in potential for use in various SUD, with mounting evidence for use of psychedelics in psychiatric conditions. If the FDA approves psilocybin and MDMA after successful phase 3 trials, there will be reduced barriers to investigate applications of psychedelics despite their current classification as Schedule I substances. Like psychedelics, but with less evidence, are neuroimmune modulating approaches to treating addiction. Without new inventions for pain treatment, new treatments for OUD and SUD which might offer the hope of a re-setting of the brain to pre-use functionality and cures we will not make the kind of progress that we need to reverse this crisis.

## Conclusion

By using agents that target pathways that lead to changes in synaptic plasticity seen in addiction, this approach can prevent addiction and/or reverse damages caused by addiction. All of these proposed approaches to treating OUD are at various stages in investigation and development. However, the potential benefits of these approaches are their ability to target structural changes that occur in the brain in addiction and treat comorbid conditions, such as other addictions and mood disorders. If successful, they will shift the paradigm of OUD treatment away from the opioid receptor and have the potential to cure, not just manage, OUD.

## Author contributions

YL: Writing – original draft, Writing – review & editing. MG: Writing – original draft, Writing – review & editing. KB: Writing – original draft, Writing – review & editing. PT: Writing – original draft, Writing – review & editing. CH: Writing – original draft, Writing – review & editing. BF: Writing – original draft, Writing – review & editing.

## References

[1] Gold MS Baron D Bowirrat A Blum K. Neurological correlates of brain reward circuitry linked to opioid use disorder (OUD): do *homo sapiens* acquire or have a reward deficiency syndrome? J Neurol Sci. (2020) 418:117137. doi: 10.1016/j.jns.2020.117137, PMID: 32957037 PMC7490287

[2] Gold MS Cadet JL Baron D Badgaiyan RD Blum K. Calvin klein (CK) designer cocktail, new “speedball” is the “grimm reaper”: brain dopaminergic surge a potential death sentence. J Syst Integr Neurosci. (2020) 7. doi: 10.15761/jsin.1000227 PMC7489280 32934822

[3] Gold MS. The role of alcohol, drugs, and deaths of despair in the U.S.'s falling life expectancy. Mo Med. (2020) 117:99–01. PMID: 32308224 PMC7144704

[4] Hill KP Gold MS Nemeroff CB McDonald W Grzenda A Widge A. Risks and benefits of Cannabis and cannabinoids in psychiatry. Am J Psychiatr. (2022) 179:98–09. doi: 10.1176/appi.ajp.2021.21030320 34875873

[5] Oesterle TS Thusius NJ Rummans TA Gold MS. Medication-assisted treatment for opioid-use disorder. Mayo Clin Proc. (2019) 94:2072–86. doi: 10.1016/j.mayocp.2019.03.029, PMID: 31543255

[6] World Drug Report (2021). Available at: https://www.unodc.org/unodc/en/data-and-analysis/wdr2021.html.

[7] Ahmad FB Cisewski JA Rossen LM Sutton P. Provisional drug overdose death counts. National Center for Health Statistics.

[8] Slavova S Rock P Bush HM Quesinberry D Walsh SL. Signal of increased opioid overdose during COVID-19 from emergency medical services data. Drug Alcohol Depend. (2020) 214:108176. doi: 10.1016/j.drugalcdep.2020.108176, PMID: 32717504 PMC7351024

[9] Alexander GC Stoller KB Haffajee RL Saloner B. An epidemic in the midst of a pandemic: opioid use disorder and COVID-19. Ann Intern Med. (2020) 173:57–8. doi: 10.7326/M20-1141, PMID: 32240283 PMC7138407

[10] Wang QQ Kaelber DC Xu R Volkow ND. COVID-19 risk and outcomes in patients with substance use disorders: analyses from electronic health records in the United States. Mol Psychiatry. (2021) 26:30–9. doi: 10.1038/s41380-020-00880-7, PMID: 32929211 PMC7488216

[11] Khatri UG Perrone J. Opioid use disorder and COVID-19: crashing of the crises. J Addict Med. (2020) 14:e6–7. doi: 10.1097/ADM.0000000000000684 32404651 PMC7236857

[12] Gold MS Deaths, despair tied to drug dependence are accelerating amid COVID-19. (2020). Available at: https://www.mdedge.com/psychiatry/article/227019/coronavirus-updates/deaths-despair-tied-drug-dependence-are-accelerating.

[13] Kariisa M Scholl L Wilson N Seth P Hoots B. Drug overdose deaths involving cocaine and psychostimulants with abuse potential - United States, 2003-2017. MMWR Morb Mortal Wkly Rep. (2019) 68:388–95. doi: 10.15585/mmwr.mm6817a3, PMID: 31048676 PMC6541315

[14] Khatri UG Viner K Perrone J. Lethal fentanyl and cocaine intoxication. N Engl J Med. (2018) 379:1782. doi: 10.1056/NEJMc1809521, PMID: 30380395

[15] Elmarasi M Garcia-Vassallo G Campbell S Fuehrlein B. Brief report: rates of fentanyl use among psychiatric emergency room patients. Am J Addict. (2021) 30:92–5. doi: 10.1111/ajad.13087, PMID: 32779217

[16] LaRue L Twillman RK Dawson E Whitley P Frasco MA Huskey A. Rate of fentanyl positivity among urine drug test results positive for cocaine or methamphetamine. JAMA Netw Open. (2019) 2:e192851. doi: 10.1001/jamanetworkopen.2019.2851, PMID: 31026029 PMC6487565

[17] Pitt AL Humphreys K Brandeau ML. Modeling health benefits and harms of public policy responses to the US opioid epidemic. Am J Public Health. (2018) 108:1394–00. doi: 10.2105/AJPH.2018.304590, PMID: 30138057 PMC6137764

[18] Gold MS. Naloxone or Narcan: Life-saving Wonder Drug (2020).

[19] Peterson C Li M Xu L Mikosz CA Luo F. Assessment of annual cost of substance use disorder in US hospitals. JAMA Netw Open. (2021) 4:e210242. doi: 10.1001/jamanetworkopen.2021.0242, PMID: 33666661 PMC7936257

[20] Strang J Volkow ND Degenhardt L Hickman M Johnson K Koob GF. Opioid use disorder. Nat Rev Dis Primers. (2020) 6:3. doi: 10.1038/s41572-019-0137-5 31919349

[21] Tsai PJ Keeley RJ Carmack SA Vendruscolo JCM Lu H Gu H. Converging structural and functional evidence for a rat salience network. Biol Psychiatry. (2020) 88:867–78. doi: 10.1016/j.biopsych.2020.06.023, PMID: 32981657

[22] Mann B Drug overdose deaths spiked to 88,000 during the pandemic, White house says. (2021). Available at: https://www.npr.org/transcripts/983414684.

[23] Connery HS Weiss RD. Discontinuing buprenorphine treatment of opioid use disorder: what do we (not) know? Am J Psychiatry. (2020) 177:104–6. doi: 10.1176/appi.ajp.2019.19121245, PMID: 32008394

[24] Bell J Strang J. Medication treatment of opioid use disorder. Biol Psychiatry. (2020) 87:82–8. doi: 10.1016/j.biopsych.2019.06.020 31420089

[25] Nunes EV Levin FR Reilly MP el-Bassel N. Medication treatment for opioid use disorder in the age of COVID-19: can new regulations modify the opioid cascade? J Subst Abus Treat. (2021) 122:108196. doi: 10.1016/j.jsat.2020.108196, PMID: 33221125 PMC7666540

[26] Srivastava AB Gold MS. Naltrexone: a history and future directions. Cerebrum. (2018) 1–14. PMC6353110 30746025

[27] Larochelle MR Bernson D Land T Stopka TJ Wang N Xuan Z. Medication for opioid use disorder after nonfatal opioid overdose and association with mortality: a cohort study. Ann Intern Med. (2018) 169:137–45. doi: 10.7326/M17-3107, PMID: 29913516 PMC6387681

[28] Bruijnzeel AW Marcinkiewcz C Isaac S Booth MM Dennis DM Gold MS. The effects of buprenorphine on fentanyl withdrawal in rats. Psychopharmacology. (2007) 191:931–41. doi: 10.1007/s00213-006-0670-2, PMID: 17211652

[29] Wolf PS Gold M. Treatment resistant opioid use disorder (TROUD): definition, rationale, and recommendations. J Neurol Sci. (2020) 411:116718. doi: 10.1016/j.jns.2020.116718 32078842

[30] Lee JD Nunes EV Novo P Bachrach K Bailey GL Bhatt S. Comparative effectiveness of XR-NTX versus buprenorphine-naloxone for opioid relapse prevention (X:BOT): a multicentre, open-label, randomised controlled trial. Lancet. (2018) 391:309–18. doi: 10.1016/S0140-6736(17)32812-X, PMID: 29150198 PMC5806119

[31] Blum K Lott L Baron D Smith D Badgaiyan R Gold M. Improving naltrexone compliance and outcomes with putative pro- dopamine regulator KB220, compared to treatment as usual. J Syst Integr Neurosci. (2020) 6:7. doi: 10.15761/JSIN.1000229 PMC7489288 32934823

[32] Downs BW Blum K Baron D Bowirrat A Lott L Brewer R. Death by opioids: are there non-addictive scientific solutions? J Syst Integr Neurosci. (2019) 5:5. doi: 10.15761/JSIN.1000211 PMC6904107 31824737

[33] Dennen AC Blum K Braverman RE Bowirrat A Gold M Elman I. How to combat the global opioid crisis. CPQ Neurol Psychol. (2023) 5 [Online ahaed of print]. PMC9937628 36812107

[34] Chen Q Larochelle MR Weaver D. Prevention of prescription opioid misuse and projected overdose deaths in the United States. Substance Use Addict. (2019) 2:1–12. doi: 10.1001/jamanetworkopen.2018.7621 PMC6415966 30707224

[35] Williams AR Samples H Crystal S Olfson M. Acute care, prescription opioid use, and overdose following discontinuation of long-term buprenorphine treatment for opioid use disorder. Am J Psychiatry. (2020) 177:117–24. doi: 10.1176/appi.ajp.2019.19060612, PMID: 31786933 PMC7002204

[36] Hill E Han D Dumouchel P Dehak N Quatieri T Moehs C. Long term suboxone™ emotional reactivity as measured by automatic detection in speech. PLoS One. (2013) 8:e69043. doi: 10.1371/journal.pone.0069043, PMID: 23874860 PMC3706486

[37] Fairley M Humphreys K Joyce VR Bounthavong M Trafton J Combs A. Cost-effectiveness of treatments for opioid use disorder. JAMA Psychiatry. (2021) 78:767–7. doi: 10.1001/jamapsychiatry.2021.0247, PMID: 33787832 PMC8014209

[38] Xu Y Wang S Chen L Shao Z Zhang M Liu S. Reduced midbrain functional connectivity and recovery in abstinent heroin users. J Psychiatr Res. (2021) 144:168–76. doi: 10.1016/j.jpsychires.2021.10.011, PMID: 34662755

[39] Blum K Liu Y Wang W Wang Y Zhang Y Oscar-Berman M. rsfMRI effects of KB220Z™ on neural pathways in reward circuitry of abstinent genotyped heroin addicts. Postgrad Med. (2015) 127:232–41. doi: 10.1080/00325481.2015.994879, PMID: 25526228 PMC4979602

[40] Swenson S Blum K McLaughlin T Gold MS Thanos PK. The therapeutic potential of exercise for neuropsychiatric diseases: a review. J Neurol Sci. (2020) 412:116763. doi: 10.1016/j.jns.2020.116763, PMID: 32305746

[41] Blum K Thompson B Demotrovics Z Femino J Giordano J Oscar-Berman M. The molecular neurobiology of twelve steps program & fellowship: connecting the dots for recovery. J Reward Defic Syndr. (2015) 1:46–4. doi: 10.17756/jrds.2015-008, PMID: 26306329 PMC4545669

[42] McLellan AT Koob GF Volkow ND. Preaddiction-a missing concept for treating substance use disorders. JAMA Psychiatry. (2022) 79:749–51. doi: 10.1001/jamapsychiatry.2022.1652 35793096

[43] Blum K Han D Bowirrat A Downs BW Bagchi D Thanos PK. Genetic addiction risk and psychological profiling analyses for "Preaddiction". Severity Index J Pers Med. (2022) 12:1–22. doi: 10.3390/jpm12111772 PMC9696872 36579510

[44] O'Donnell JK Gladden RM Seth P. Trends in deaths involving heroin and synthetic opioids excluding methadone, and law enforcement drug product reports, by census region - United States, 2006-2015. MMWR Morb Mortal Wkly Rep. (2017) 66:897–03. doi: 10.15585/mmwr.mm6634a2, PMID: 28859052 PMC5657786

[45] Renard J Rosen LG Loureiro M de Oliveira C Schmid S Rushlow WJ. Adolescent cannabinoid exposure induces a persistent sub-cortical hyper-dopaminergic state and associated molecular adaptations in the prefrontal cortex. Cereb Cortex. (2017) 27:1297–10. doi: 10.1093/cercor/bhv335, PMID: 26733534

[46] Edwards D Roy Boyett B Badgaiyan RD Thanos PK Baron D. Addiction by any other name is still addiction: embracing molecular neurogenetic/epigenetic basis of reward deficiency. J Addict Sci. (2020) 6:1–4. doi: 10.17756/jas.2020-043, PMID: 32432229 PMC7236379

[47] Gondré-Lewis MC Bassey R Blum K. Pre-clinical models of reward deficiency syndrome: a behavioral octopus. Neurosci Biobehav Rev. (2020) 115:164–88. doi: 10.1016/j.neubiorev.2020.04.021, PMID: 32360413 PMC7594013

[48] Mauro PM Gutkind S Annunziato EM Samples H. Use of medication for opioid use disorder among US adolescents and adults with need for opioid treatment, 2019. JAMA Netw Open. (2022) 5:e223821. doi: 10.1001/jamanetworkopen.2022.3821, PMID: 35319762 PMC8943638

[49] Treitler PC Bowden CF Lloyd J Enich M Nyaku AN Crystal S. Perspectives of opioid use disorder treatment providers during COVID-19: adapting to flexibilities and sustaining reforms. J Subst Abus Treat. (2022) 132:108514. doi: 10.1016/j.jsat.2021.108514, PMID: 34098210 PMC8630075

[50] Olsen Y Fitzgerald RM Wakeman SE. Overcoming barriers to treatment of opioid use disorder. JAMA. (2021) 325:1149–50. doi: 10.1001/jama.2021.1741 33630021

[51] Weimer MB Wakeman SE Saitz R. Removing one barrier to opioid use disorder treatment: is it enough? JAMA. (2021) 325:1147–8. doi: 10.1001/jama.2021.0958, PMID: 33630020

[52] Hawk K Hoppe J Ketcham E LaPietra A Moulin A Nelson L. Consensus recommendations on the treatment of opioid use disorder in the emergency department. Ann Emerg Med. (2021) 78:434–42. doi: 10.1016/j.annemergmed.2021.04.023, PMID: 34172303

[53] Herring AA Vosooghi AA Luftig J Anderson ES Zhao X Dziura J. High-dose buprenorphine induction in the emergency Department for Treatment of opioid use disorder. JAMA Netw Open. (2021) 4:e2117128. doi: 10.1001/jamanetworkopen.2021.17128, PMID: 34264326 PMC8283555

[54] Blum K Jacobs W Modestino EJ DiNubile N Baron D McLaughlin T. Insurance companies fighting the peer review empire without any validity: the case for addiction and pain modalities in the face of an American drug epidemic. SEJ Surg Pain. (2018) 1:1–11. PMID: 29911684 PMC5998670

[55] Krawczyk N. Has the treatment gap for opioid use disorder narrowed in the U.S.?: a yearly assessment from 2010 to 2019. Int J Drug Policy. (2022) 110:103786 35934583 10.1016/j.drugpo.2022.103786 PMC10976290

[56] Taylor JL Johnson S Cruz R Gray JR Schiff D Bagley SM. Integrating harm reduction into outpatient opioid use disorder treatment settings: harm reduction in outpatient addiction treatment. J Gen Intern Med. (2021) 36:3810–9. doi: 10.1007/s11606-021-06904-4, PMID: 34159545 PMC8218967

[57] Dackis CA Gold MS. New concepts in cocaine addiction: the dopamine depletion hypothesis. Neurosci Biobehav Rev. (1985) 9:469–77. doi: 10.1016/0149-7634(85)90022-3, PMID: 2999657

[58] Blum K Bowirrat A Gomez LL Downs BW Bagchi D Barh D. Why haven't we solved the addiction crisis? J Neurol Sci. (2022) 442:120404. doi: 10.1016/j.jns.2022.120404, PMID: 36084363

[59] Febo M Blum K Badgaiyan RD Baron D Thanos PK Colon-Perez LM. Dopamine homeostasis: brain functional connectivity in reward deficiency syndrome. Front Biosci. (2017) 22:669–91. doi: 10.2741/4509, PMID: 27814639

[60] Cheron J Kerchove d'Exaerde A. Drug addiction: from bench to bedside. Transl Psychiatry. (2021) 11:424. doi: 10.1038/s41398-021-01542-0 34385417 PMC8361217

[61] Koob GF Volkow ND. Neurobiology of addiction: a neurocircuitry analysis. Lancet Psychiatry. (2016) 3:760–73. doi: 10.1016/S2215-0366(16)00104-8, PMID: 27475769 PMC6135092

[62] Luigjes J Segrave R de Joode N Figee M Denys D. Efficacy of invasive and non-invasive brain modulation interventions for addiction. Neuropsychol Rev. (2019) 29:116–38. doi: 10.1007/s11065-018-9393-5, PMID: 30536145 PMC6499746

[63] Wang W Zhu Y Wang L Mu LL Zhu L Ding D. High-frequency repetitive transcranial magnetic stimulation of the left dorsolateral prefrontal cortex reduces drug craving and improves decision-making ability in methamphetamine use disorder. Psychiatry Res. (2022) 317:114904. doi: 10.1016/j.psychres.2022.114904, PMID: 36265196

[64] Li X Hartwell KJ Owens M LeMatty T Borckardt JJ Hanlon CA. Repetitive transcranial magnetic stimulation of the dorsolateral prefrontal cortex reduces nicotine cue craving. Biol Psychiatry. (2013) 73:714–20. doi: 10.1016/j.biopsych.2013.01.003, PMID: 23485014 PMC3615051

[65] Ekhtiari H Tavakoli H Addolorato G Baeken C Bonci A Campanella S. Transcranial electrical and magnetic stimulation (tES and TMS) for addiction medicine: a consensus paper on the present state of the science and the road ahead. Neurosci Biobehav Rev. (2019) 104:118–40. doi: 10.1016/j.neubiorev.2019.06.007, PMID: 31271802 PMC7293143

[66] Dinur-Klein L Dannon P Hadar A Rosenberg O Roth Y Kotler M. Smoking cessation induced by deep repetitive transcranial magnetic stimulation of the prefrontal and insular cortices: a prospective, randomized controlled trial. Biol Psychiatry. (2014) 76:742–9. doi: 10.1016/j.biopsych.2014.05.020 25038985

[67] Addolorato G Antonelli M Cocciolillo F Vassallo GA Tarli C Sestito L. Deep transcranial magnetic stimulation of the dorsolateral prefrontal cortex in alcohol use disorder patients: effects on dopamine transporter availability and alcohol intake. Eur Neuropsychopharmacol. (2017) 27:450–61. doi: 10.1016/j.euroneuro.2017.03.008, PMID: 28390775

[68] Prikryl R Ustohal L Kucerova HP Kasparek T Jarkovsky J Hublova V. Repetitive transcranial magnetic stimulation reduces cigarette consumption in schizophrenia patients. Prog Neuro-Psychopharmacol Biol Psychiatry. (2014) 49:30–5. doi: 10.1016/j.pnpbp.2013.10.019, PMID: 24211840

[69] Terraneo A Leggio L Saladini M Ermani M Bonci A Gallimberti L. Transcranial magnetic stimulation of dorsolateral prefrontal cortex reduces cocaine use: a pilot study. Eur Neuropsychopharmacol. (2016) 26:37–44. doi: 10.1016/j.euroneuro.2015.11.011, PMID: 26655188 PMC9379076

[70] Liu X Zhao X Liu T Liu Q Tang L Zhang H. The effects of repetitive transcranial magnetic stimulation on cue-induced craving in male patients with heroin use disorder. EBioMedicine. (2020) 56:102809. doi: 10.1016/j.ebiom.2020.102809, PMID: 32512513 PMC7276507

[71] Gay A Cabe J de Chazeron I Lambert C Defour M Bhoowabul V. Repetitive transcranial magnetic stimulation (rTMS) as a promising treatment for craving in stimulant drugs and Behavioral addiction: a Meta-analysis. J Clin Med. (2022) 11:1–27. doi: 10.3390/jcm11030624, PMID: 35160085 PMC8836499

[72] Sahlem G (2023). Accelerated intermittent Theta-burst stimulation for opiate use disorder. Available at: https://clinicaltrials.gov/ct2/show/NCT03804619?term=TMS&cond=Opioid+Use&draw=3&rank=18.

[73] Lee H (2022). Repetitive transcranial magnetic stimulation to reduce heroin cravings U.S. National Library of Medicine. Available at: https://clinicaltrials.gov/ct2/show/NCT05074524?term=TMS&cond=Opioid+Use&draw=3&rank=14.

[74] Hanlon C (2023). H-coil TMS to reduce pain: a pilot study evaluating relative efficacy of the H1 vs H7 coil. Available at: https://clinicaltrials.gov/ct2/show/NCT04203199?term=TMS&cond=Opioid+Use&draw=3&rank=13.

[75] Voineskos D (2022). rTMS for suicidality in opioid use disorder. Available at: https://clinicaltrials.gov/ct2/show/NCT04785456?term=TMS&cond=Opioid+Use&draw=3&rank=11.

[76] Greenwald M (2023). rTMS, stress and opioid use disorder (TOTS) Available at: https://clinicaltrials.gov/ct2/show/NCT04231708?term=TMS&cond=Opioid+Use&draw=2&rank=8.

[77] Greenwald M (2023). Effects of pharmacological stress and rTMS on executive function in opioid use Disorde. Available at: https://clinicaltrials.gov/ct2/show/NCT04231708?term=TMS&cond=Opioid+Use&draw=2&rank=8.

[78] Rush C (2023). Adjunctive transcranial stimulation to reduce impulsivity in opiate use disorder. Available at: https://clinicaltrials.gov/ct2/show/NCT05049460?term=TMS&cond=Opioid+Use&draw=2&rank=7

[79] Wang T-Y (2020). Repetitive transcranial magnetic stimulation in patients with opioid use disorders. Available at: https://clinicaltrials.gov/ct2/show/NCT03229642?term=TMS&cond=Opioid+Use&draw=2&rank=6.

[80] Haroun A (2022). The role of repetitive trans cranial-magnetic stimulation in craving reduction among opioid use disorder patients. Available at: https://clinicaltrials.gov/ct2/show/NCT04691167?term=TMS&cond=Opioid+Use&draw=2&rank=5

[81] Mahoney J (2023). An open-label trial of repetitive transcranial magnetic stimulation for opioid use disorder. Available at: https://clinicaltrials.gov/ct2/show/NCT04157062?term=TMS&cond=Opioid+Use&draw=2&rank=4.

[82] Biernacki K (2023). Using combined EEG and non-invasive brain stimulation to examine and improve reward functioning in opioid use disorder. Available at: https://clinicaltrials.gov/ct2/show/NCT04432493?term=TMS&cond=Opioid+Use&draw=2&rank=3

[83] Mcrae-Clark AL (2022). Transcranial magnetic stimulation (rTMS) as a tool to decrease pain and improve functioning (TMS). Available at: https://clinicaltrials.gov/ct2/show/NCT03821337?term=TMS&cond=Opioid+Use&draw=2&rank=2.

[84] Steele VR Maxwell AM. Treating cocaine and opioid use disorder with transcranial magnetic stimulation: a path forward. Pharmacol Biochem Behav. (2021) 209:173240. doi: 10.1016/j.pbb.2021.173240, PMID: 34298030 PMC8445657

[85] Taghva A Silvetz R Ring A Kim KYA Murphy KT Liu CY. Magnetic resonance therapy improves clinical phenotype and EEG alpha power in posttraumatic stress disorder. Trauma Mon. (2015) 20:e27360. doi: 10.5812/traumamon.27360 26839865 PMC4727473

[86] Hassan O Phan S Wiecks N Joaquin C Bondarenko V. Outcomes of deep brain stimulation surgery for substance use disorder: a systematic review. Neurosurg Rev. (2021) 44:1967–76. doi: 10.1007/s10143-020-01415-y 33037538

[87] Brown J (2023). Temporal interference neurostimulation and addiction. Available at: https://clinicaltrials.gov/ct2/show/NCT04432064?term=DBS&cond=Addiction&draw=2&rank=10.

[88] Sun B (2020). Deep brain stimulation of the bilateral nucleus Accumbens for patients with methadone maintenance treatment. Available at: https://clinicaltrials.gov/ct2/show/NCT03952455?term=DBS&cond=Addiction&draw=2&rank=7

[89] Lu L (2018). Deep brain stimulation of nucleus Accumbens for opioid relapse prevention. Available at: https://clinicaltrials.gov/ct2/show/NCT03424616?term=DBS&cond=Addiction&draw=2&rank=6

[90] Luming L (2016). PINS stimulator system for deep brain stimulation of the nucleus Accumbens to treat severe opioid addiction. Available at: https://clinicaltrials.gov/ct2/show/NCT02282072?term=DBS&cond=Addiction&draw=2&rank=5

[91] Kuhn VV. (2016). Deep brain stimulation of the nucleus Accumbens as a novel treatment in severe opioid addiction (NASA). Available at: https://clinicaltrials.gov/ct2/show/NCT01245075?term=DBS&cond=Addiction&draw=2&rank=4 10.1038/mp.2012.196 23337942

[92] Tomycz N (2023). Deep brain stimulation effects in patients with opioid use disorder. Available at: https://clinicaltrials.gov/ct2/show/NCT04354077?term=DBS&cond=Addiction&draw=3&rank=13.

[93] Gao G-D (2016). Brain electrophysiological study(EEG/ERP) on opiate addicts treating by bilateral NAc/ALIC deep brain stimulation. Available at: https://clinicaltrials.gov/ct2/show/NCT02594306?term=DBS&cond=Addiction&draw=3&rank=15.

[94] Gao G-D (2014). Deep brain stimulation of nucleus Accumbens to prevent opiate relapse. Available at: https://clinicaltrials.gov/ct2/show/NCT01274988?term=DBS&cond=Addiction&draw=2&rank=8

[95] Elias WJ Lipsman N Ondo WG Ghanouni P Kim YG Lee W. A randomized trial of focused ultrasound thalamotomy for essential tremor. N Engl J Med. (2016) 375:730–9. doi: 10.1056/NEJMoa1600159 27557301

[96] Bond AE Shah BB Huss DS Dallapiazza RF Warren A Harrison MB. Safety and efficacy of focused ultrasound thalamotomy for patients with medication-refractory, tremor-dominant Parkinson disease: a randomized clinical trial. JAMA Neurol. (2017) 74:1412–8. doi: 10.1001/jamaneurol.2017.3098, PMID: 29084313 PMC5822192

[97] Razza LB Palumbo P Moffa AH Carvalho AF Solmi M Loo CK. A systematic review and meta-analysis on the effects of transcranial direct current stimulation in depressive episodes. Depress Anxiety. (2020) 37:594–08. doi: 10.1002/da.23004, PMID: 32101631

[98] Thair H Holloway AL Newport R Smith AD. Transcranial direct current stimulation (tDCS): a Beginner's guide for design and implementation. Front Neurosci. (2017) 11:641. doi: 10.3389/fnins.2017.00641, PMID: 29213226 PMC5702643

[99] Stagg CJ Nitsche MA. Physiological basis of transcranial direct current stimulation. Neuroscientist. (2011) 17:37–53. doi: 10.1177/1073858410386614 21343407

[100] Brunoni AR Nitsche MA Bolognini N Bikson M Wagner T. Clinical research with transcranial direct current stimulation (tDCS): challenges and future directions. Brain Stimul. (2012) 5:175–95. doi: 10.1016/j.brs.2011.03.002, PMID: 22037126 PMC3270156

[101] Nitsche MA Cohen LG Wassermann EM Priori A Lang N Antal A. Transcranial direct current stimulation: state of the art 2008. Brain Stimul. (2008) 1:206–23. doi: 10.1016/j.brs.2008.06.004, PMID: 20633386

[102] InSightec. (2023). Exablate for LIFU neuromodulation in patients with opioid use disorder (OUD). Available at: https://clinicaltrials.gov/ct2/show/NCT04197921?term=focused+ultrasound&cond=opioid&draw=2&rank=1.

[103] Nutt D Carhart-Harris R. The current status of psychedelics in psychiatry. JAMA Psychiatry. (2021) 78:121–2. doi: 10.1001/jamapsychiatry.2020.2171, PMID: 32725172

[104] Nichols D Johnson M Nichols C. Psychedelics as medicines: an emerging new paradigm. Clin Pharmacol Therap. (2017) 101:209–19. doi: 10.1002/cpt.557, PMID: 28019026

[105] Abdallah CG Krystal JH. Ketamine and rapid acting antidepressants: are we ready to cure, rather than treat depression? Behav Brain Res. (2020) 390:112628. doi: 10.1016/j.bbr.2020.112628, PMID: 32407817 PMC7316409

[106] Fantegrossi WE Murnane KS Reissig CJ. The behavioral pharmacology of hallucinogens. Biochem Pharmacol. (2008) 75:17–33. doi: 10.1016/j.bcp.2007.07.018, PMID: 17977517 PMC2247373

[107] Rodrigues LS Rossi GN Rocha JM Osório F Bouso JC Hallak JEC. Effects of ayahuasca and its alkaloids on substance use disorders: an updated (2016-2020) systematic review of preclinical and human studies. Eur Arch Psychiatry Clin Neurosci. (2022) 272:541–56. doi: 10.1007/s00406-021-01267-7, PMID: 33914164

[108] Shao LX Liao C Gregg I Davoudian PA Savalia NK Delagarza K. Psilocybin induces rapid and persistent growth of dendritic spines in frontal cortex in vivo. Neuron. (2021) 109:2535–2544.e4. doi: 10.1016/j.neuron.2021.06.008, PMID: 34228959 PMC8376772

[109] Ly C Greb AC Cameron LP Wong JM Barragan EV Wilson PC. Psychedelics promote structural and functional neural plasticity. Cell Rep. (2018) 23:3170–82. doi: 10.1016/j.celrep.2018.05.022, PMID: 29898390 PMC6082376

[110] Daly EJ Trivedi MH Janik A Li H Zhang Y Li X. Efficacy of Esketamine nasal spray plus Oral antidepressant treatment for relapse prevention in patients with treatment-resistant depression: a randomized clinical trial. JAMA Psychiatry. (2019) 76:893–03. doi: 10.1001/jamapsychiatry.2019.1189, PMID: 31166571 PMC6551577

[111] Popova V Daly EJ Trivedi M Cooper K Lane R Lim P. Efficacy and safety of flexibly dosed Esketamine nasal spray combined with a newly initiated Oral antidepressant in treatment-resistant depression: a randomized double-blind active-controlled study. Am J Psychiatry. (2019) 176:428–38. doi: 10.1176/appi.ajp.2019.19020172, PMID: 31109201

[112] Marcus DJ Bruchas MR. Where ketamine and dopamine collide. Elife. (2021) 10:148. doi: 10.7554/eLife.70148 PMC8211445 34137373

[113] Awakn Awakn announces positive results from phase II a/B clinical trial. Awakn life sciences Corp. (2022).

[114] COMPASS Pathways announces positive topline results from groundbreaking phase IIb trial of investigational COMP360 psilocybin therapy for treatment-resistant depression. (2021). Available at: https://ir.compasspathways.com/news-releases/news-release-details/compass-pathways-announces-positive-topline-results.

[115] Fischer S. Psilocybin therapy in advanced Cancer. Available at: https://clinicaltrials.gov/ct2/show/NCT05398484?term=psilocybin&phase=2&draw=2&rank=1.

[116] Smith KW Sicignano DJ Hernandez AV White CM. MDMA-assisted psychotherapy for treatment of posttraumatic stress disorder: a systematic review with Meta-analysis. J Clin Pharmacol. (2022) 62:463–71. doi: 10.1002/jcph.1995, PMID: 34708874

[117] Mitchell JM Bogenschutz M Lilienstein A Harrison C Kleiman S Parker-Guilbert K. MDMA-assisted therapy for severe PTSD: a randomized, double-blind, placebo-controlled phase 3 study. Nat Med. (2021) 27:1025–33. doi: 10.1038/s41591-021-01336-3, PMID: 33972795 PMC8205851

[118] MAPP2 (2023). A multi-site phase 3 study of MDMA-assisted therapy for PTSD (MAPP2) Available at: https://maps.org/mdma/ptsd/mapp2/.

[119] Garcia-Romeu A Davis AK Erowid E Erowid F Griffiths RR Johnson MW. Persisting reductions in Cannabis, opioid, and stimulant misuse after naturalistic psychedelic use: an online survey. Front Psych. (2019) 10:955. 10.3389/fpsyt.2019.00955 PMC6987443 32038317

[120] Bogenschutz MP Forcehimes AA Pommy JA Wilcox CE Barbosa PCR Strassman RJ. Psilocybin-assisted treatment for alcohol dependence: a proof-of-concept study. J Psychopharmacol. (2015) 29:289–99. doi: 10.1177/0269881114565144, PMID: 25586396

[121] Johnson MW Garcia-Romeu A Cosimano MP Griffiths RR. Pilot study of the 5-HT2AR agonist psilocybin in the treatment of tobacco addiction. J Psychopharmacol. (2014) 28:983–92. doi: 10.1177/0269881114548296, PMID: 25213996 PMC4286320

[122] Johnson MW Garcia-Romeu A Griffiths RR. Long-term follow-up of psilocybin-facilitated smoking cessation. Am J Drug Alcohol Abuse. (2017) 43:55–60. doi: 10.3109/00952990.2016.1170135, PMID: 27441452 PMC5641975

[123] Walsh Z Mollaahmetoglu OM Rootman J Golsof S Keeler J Marsh B. Ketamine for the treatment of mental health and substance use disorders: comprehensive systematic review. BJPsych Open. (2021) 8:e19. doi: 10.1192/bjo.2021.1061 35048815 PMC8715255

[124] Khawaja H (2023). MDMA for co-occurring PTSD and OUD after childbirth. Available at: https://clinicaltrials.gov/ct2/show/NCT05219175?term=psychedelic&cond=opioid&draw=2&rank=1.

[125] Garland E (2023). Ketamine assisted psychotherapy for opioid use disorder. Available at: https://clinicaltrials.gov/ct2/show/NCT04892251?term=ketamine&cond=opioid+use&draw=2&rank=1

[126] Jones J (2023). Ketamine for the treatment of opioid use disorder and depression. Available at: https://clinicaltrials.gov/ct2/show/NCT04177706?term=ketamine&cond=opioid+use&draw=2&rank=2.

[127] Belcher A (2023). Ketamine for OUD and comorbid depression (OUDCD). U.S. National Library of Medicine Available at: https://clinicaltrials.gov/ct2/show/NCT05051449?term=ketamine&cond=opioid+use&draw=2&rank=5.

[128] Brown R (2023). Adjunctive effects of psilocybin and buprenorphine. Available at: https://clinicaltrials.gov/ct2/show/NCT04161066?term=psychedelic&cond=opioid&draw=2&rank=4.

[129] Johnson MW (2023). Psilocybin for opioid use disorder in patients on methadone maintenance with ongoing opioid use. Available at: https://clinicaltrials.gov/ct2/show/NCT05242029?term=psychedelic&cond=opioid&draw=2&rank=3e.

[130] Louw WF (2023). Standardized natural psilocybin-assisted psychotherapy for tapering of opioid medication. Available at: https://clinicaltrials.gov/ct2/show/NCT05585229?term=psychedelic&cond=opioid&draw=2&rank=2.

[131] Palhano-Fontes F Barreto D Onias H Andrade KC Novaes MM Pessoa JA. Rapid antidepressant effects of the psychedelic ayahuasca in treatment-resistant depression: a randomized placebo-controlled trial. Psychol Med. (2019) 49:655–63. doi: 10.1017/S0033291718001356, PMID: 29903051 PMC6378413

[132] Agin-Liebes G Haas TF Lancelotta R Uthaug MV Ramaekers JG Davis AK. Naturalistic use of mescaline is associated with self-reported psychiatric improvements and enduring positive life changes. ACS Pharmacol Transl Sci. (2021) 4:543–52. doi: 10.1021/acsptsci.1c00018, PMID: 33860184 PMC8033766

[133] Thomas G Lucas P Capler N Tupper K Martin G. Ayahuasca-assisted therapy for addiction: results from a preliminary observational study in Canada. Curr Drug Abuse Rev. (2013) 6:30–42. doi: 10.2174/15733998113099990003, PMID: 23627784

[134] Bogenschutz MP Johnson MW. Classic hallucinogens in the treatment of addictions. Prog Neuro-Psychopharmacol Biol Psychiatry. (2016) 64:250–8. doi: 10.1016/j.pnpbp.2015.03.002 25784600

[135] Brown TK Alper K. Treatment of opioid use disorder with ibogaine: detoxification and drug use outcomes. Am J Drug Alcohol Abuse. (2018) 44:24–36. doi: 10.1080/00952990.2017.1320802 28541119

[136] Wilson C Millar T Matieschyn Z. Novel treatment of opioid use disorder using ibogaine and iboga in two adults. J Psychedel Stud. (2020) 4:1–7. doi: 10.1556/2054.2020.00133

[137] Malcolm BJ Polanco M Barsuglia JP. Changes in withdrawal and craving scores in participants undergoing opioid detoxification utilizing ibogaine. J Psychoactive Drugs. (2018) 50:256–65. doi: 10.1080/02791072.2018.1447175, PMID: 29608409

[138] Mash DC Kovera CA Pablo J Tyndale R Ervin FR Kamlet JD. Ibogaine in the treatment of heroin withdrawal. Alkaloids Chem Biol. (2001) 56:155–71. doi: 10.1016/S0099-9598(01)56012-5 11705106

[139] Brown TK. Ibogaine in the treatment of substance dependence. Curr Drug Abuse Rev. (2013) 6:3–16. doi: 10.2174/15672050113109990001 23627782

[140] Krebs TS Johansen P. Lysergic acid diethylamide (LSD) for alcoholism: meta-analysis of randomized controlled trials. J Psychopharmacol. (2012) 26:994–02. doi: 10.1177/0269881112439253, PMID: 22406913

[141] Blanco C Volkow ND. Management of opioid use disorder in the USA: present status and future directions. Lancet. (2019) 393:1760–72. doi: 10.1016/S0140-6736(18)33078-2, PMID: 30878228

[142] Namba MD Leyrer-Jackson JM Nagy EK Olive MF Neisewander JL. Neuroimmune mechanisms as novel treatment targets for substance use disorders and associated comorbidities. Front Neurosci. (2021) 15:650785. doi: 10.3389/fnins.2021.650785, PMID: 33935636 PMC8082184

[143] Moussawi K Pacchioni A Moran M Olive MF Gass JT Lavin A. N-acetylcysteine reverses cocaine-induced metaplasticity. Nat Neurosci. (2009) 12:182–9. doi: 10.1038/nn.2250, PMID: 19136971 PMC2661026

[144] Knackstedt LA Melendez RI Kalivas PW. Ceftriaxone restores glutamate homeostasis and prevents relapse to cocaine seeking. Biol Psychiatry. (2010) 67:81–4. doi: 10.1016/j.biopsych.2009.07.018, PMID: 19717140 PMC2795043

[145] Abulseoud OA Camsari UM Ruby CL Kasasbeh A Choi S Choi DS. Attenuation of ethanol withdrawal by ceftriaxone-induced upregulation of glutamate transporter EAAT2. Neuropsychopharmacology. (2014) 39:1674–84. doi: 10.1038/npp.2014.14, PMID: 24452391 PMC4023140

[146] Farokhnia M Rentsch CT Chuong V McGinn MA Elvig SK Douglass EA. Spironolactone as a potential new pharmacotherapy for alcohol use disorder: convergent evidence from rodent and human studies. Mol Psychiatry. (2022) 27:4642–52. doi: 10.1038/s41380-022-01736-y, PMID: 36123420 PMC10231646

[147] Bachtell R Hutchinson M Wang X Rice K Maier S Watkins L. Targeting the toll of drug abuse: the translational potential of toll-like receptor 4. CNS Neurol Disord Drug Targets. (2015) 14:692–9. doi: 10.2174/1871527314666150529132503, PMID: 26022268 PMC5548122

[148] Wang X Northcutt AL Cochran TA Zhang X Fabisiak TJ Haas ME. Methamphetamine activates toll-like receptor 4 to induce central immune Signaling within the ventral tegmental area and contributes to extracellular dopamine increase in the nucleus Accumbens Shell. ACS Chem Neurosci. (2019) 10:3622–34. doi: 10.1021/acschemneuro.9b00225, PMID: 31282647 PMC8316980

[149] Hutchinson MR Northcutt AL Hiranita T Wang X Lewis SS Thomas J. Opioid activation of toll-like receptor 4 contributes to drug reinforcement. J Neurosci. (2012) 32:11187–200. doi: 10.1523/JNEUROSCI.0684-12.2012, PMID: 22895704 PMC3454463

[150] Truong TT Kosten TR. Current status of vaccines for substance use disorders: a brief review of human studies. J Neurol Sci. (2022) 434:120098. doi: 10.1016/j.jns.2021.120098 34952345

[151] Haney M Gunderson EW Jiang H Collins ED Foltin RW. Cocaine-specific antibodies blunt the subjective effects of smoked cocaine in humans. Biol Psychiatry. (2010) 67:59–65. doi: 10.1016/j.biopsych.2009.08.031, PMID: 19846066 PMC3319755

[152] Ohia-Nwoko O Kosten TA Haile CN. Animal models and the development of vaccines to treat substance use disorders. Int Rev Neurobiol. (2016) 126:263–91. doi: 10.1016/bs.irn.2016.02.009, PMID: 27055616

[153] Xu A Kosten TR. Current status of immunotherapies for addiction. Ann N Y Acad Sci. (2021) 1489:3–16. doi: 10.1111/nyas.14329, PMID: 32147860

[154] Pravetoni M. Biologics to treat substance use disorders: current status and new directions. Hum Vaccin Immunother. (2016) 12:3005–19. doi: 10.1080/21645515.2016.1212785, PMID: 27441896 PMC5215474

